# Pathological complete response in microsatellite- stable gastric cancer with liver and bulky lymph node metastases after nivolumab-based chemotherapy and surgery: a case report

**DOI:** 10.3389/fimmu.2026.1750532

**Published:** 2026-02-18

**Authors:** Kenichi Nonaka, Koji Maniwa, Chika Takao, Minoru Komura, Yoshinori Mushika, Noriyuki Takeuchi, Toshio Kato, Arizumi Kikuchi, Mitsuhiko Kusakabe

**Affiliations:** 1 Department of Digestive Surgery, Daiyukai General Hospital, Ichinomiya, Japan; 2 Department of Surgery, Daiyukai General Hospital, Ichinomiya, Japan; 3 Department of Pathology, Daiyukai General Hospital, Ichinomiya, Japan; 4 Daiyukai Research Institute for Medical Science, Ichinomiya, Japan

**Keywords:** bulky lymph node metastasis, gastric cancer, immune checkpoint inhibitor, liver metastasis, microsatellite stable, pathological complete response, PD-L1

## Abstract

**Background:**

Gastric cancer with liver metastases is generally associated with poor prognosis, and curative treatment is rarely achieved. Recently, the combination of cytotoxic chemotherapy and immune checkpoint inhibitors (ICIs) has shown promise. However, the efficacy of ICIs in treating microsatellite-stable (MSS) gastric cancer remains controversial. Herein, we present a case of advanced MSS gastric cancer with liver and bulky lymph node metastases, in which combination therapy with S-1 plus oxaliplatin (SOX) and nivolumab led to a pathological complete response.

**Case presentation:**

A 64-year-old man was diagnosed with gastric adenocarcinoma with a solitary liver metastasis (S6) and bulky regional lymph node metastasis. Four cycles of SOX and nivolumab (360 mg every 3 weeks) were administered. Imaging revealed marked tumor regression, and surgery was performed. The patient underwent distal gastrectomy with D2 lymphadenectomy and partial hepatectomy. Pathological evaluation revealed complete tumor regression in the primary lesion, lymph nodes, and liver. The tumor was MSS, Epstein–Barr virus-negative and had a low tumor mutational burden (TMB). Immunohistochemistry showed a mean CD8+ tumor-infiltrating lymphocyte density of 59.5 ± 14.3 cells per high-power field, a programmed death-ligand 1 (PD-L1) combined positive score (CPS) of ≥ 70% (clone 28-8), and a germline TP53 p.R106C mutation.

**Conclusion:**

This case illustrates that even in MSS gastric cancer with low TMB levels, exceptionally high PD-L1 expression may predict a profound response to ICI-based therapy. The PD-L1 CPS may serve as a critical biomarker independent of TMB or microsatellite-instability status.

## 1 Introduction

Gastric cancer with synchronous liver metastasis has traditionally been considered unresectable due to poor outcomes and high postoperative recurrence rates. Even in cases of solitary metastases, the probability of systemic micrometastases limits the role of curative resections. Recent developments in systemic therapies, particularly the introduction of immune checkpoint inhibitors (ICIs), have shifted this treatment paradigm.

Nivolumab, a monoclonal antibody targeting programmed cell death protein 1, has shown clinical benefits in advanced gastric cancer, particularly in tumors that are microsatellite-instability (MSI)-high (MSI-H) or have a programmed death-ligand 1 (PD-L1) combined positive score (CPS) ≥ 5%. However, the efficacy of ICIs in treating microsatellite-stable (MSS) tumors remains unclear. Herein, we report a case of advanced MSS gastric cancer with liver and bulky lymph node metastases that achieved a pathological complete response (pCR) after S-1 plus oxaliplatin (SOX) and nivolumab therapy. This finding suggests that even in MSS gastric cancer, immune checkpoint therapy can be remarkably effective in cases with high PD-L1 expression.

## 2 Case presentation

**Figure 1:**
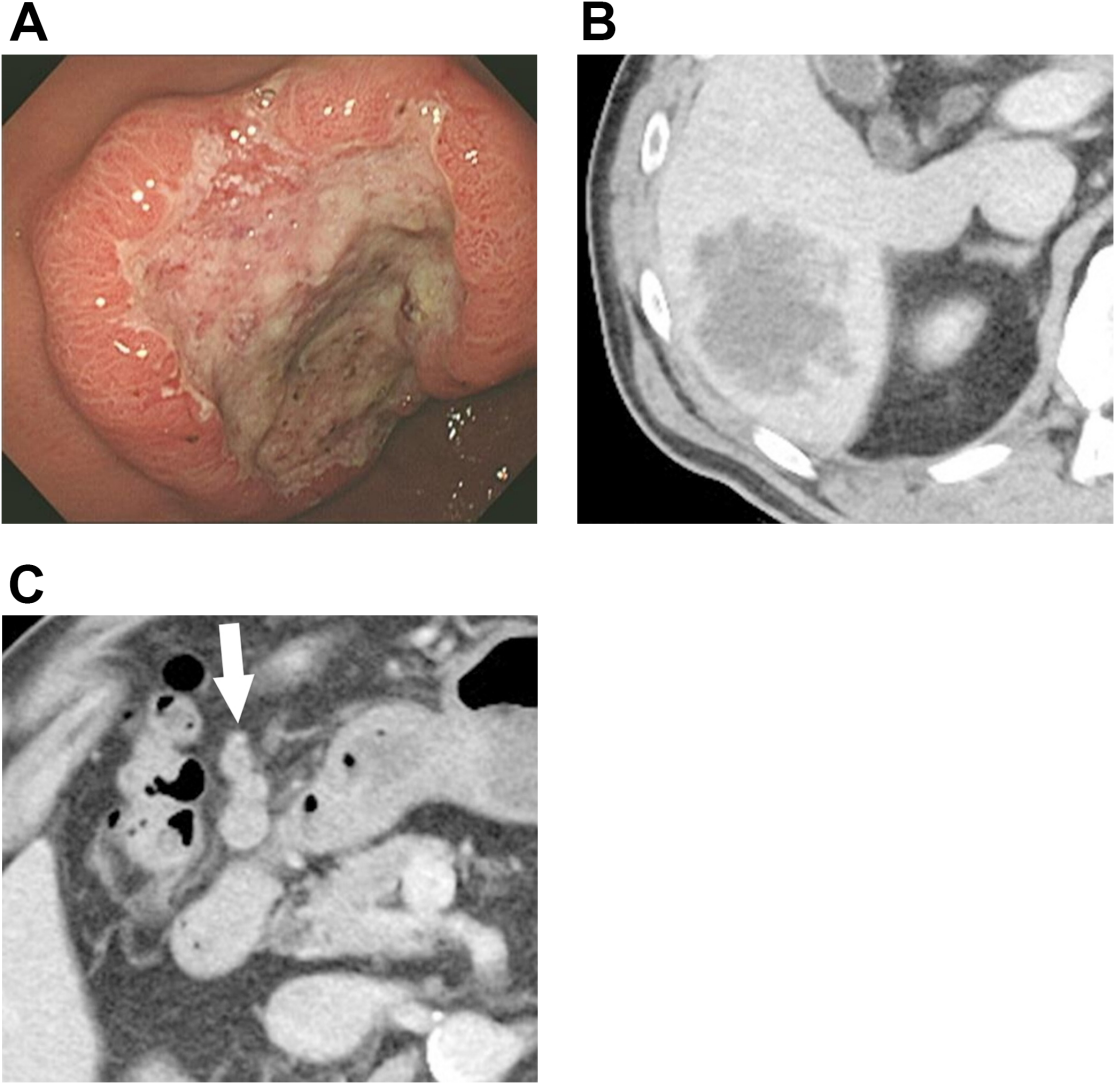
Findings from pre-chemotherapy examinations. **(A)** Endoscopy reveals a type 2 lesion, approximately 5 cm in diameter, located on the lesser curvature of the antrum. **(B)** Initial CT reveals an approximately 6-cm metastatic lesion in liver segment six with irregular margins and heterogeneous internal density. **(C)** At the initial presentation, multiple enlarged perigastric lymph nodes are observed, suggestive of lymph node metastasis (white arrow). CT, computed tomography.

A 64-year-old man presented to our hospital with epigastric pain. Endoscopy revealed a type 2 tumor in the lesser curvature of the antrum (**Figure 1A**). Biopsy confirmed a moderately differentiated adenocarcinoma. Computed tomography (CT) revealed a 6-cm S6 liver metastasis (**Figure 1B**) and multiple bulky regional lymph node enlargements (**Figure 1C**).

**Figure 2:**
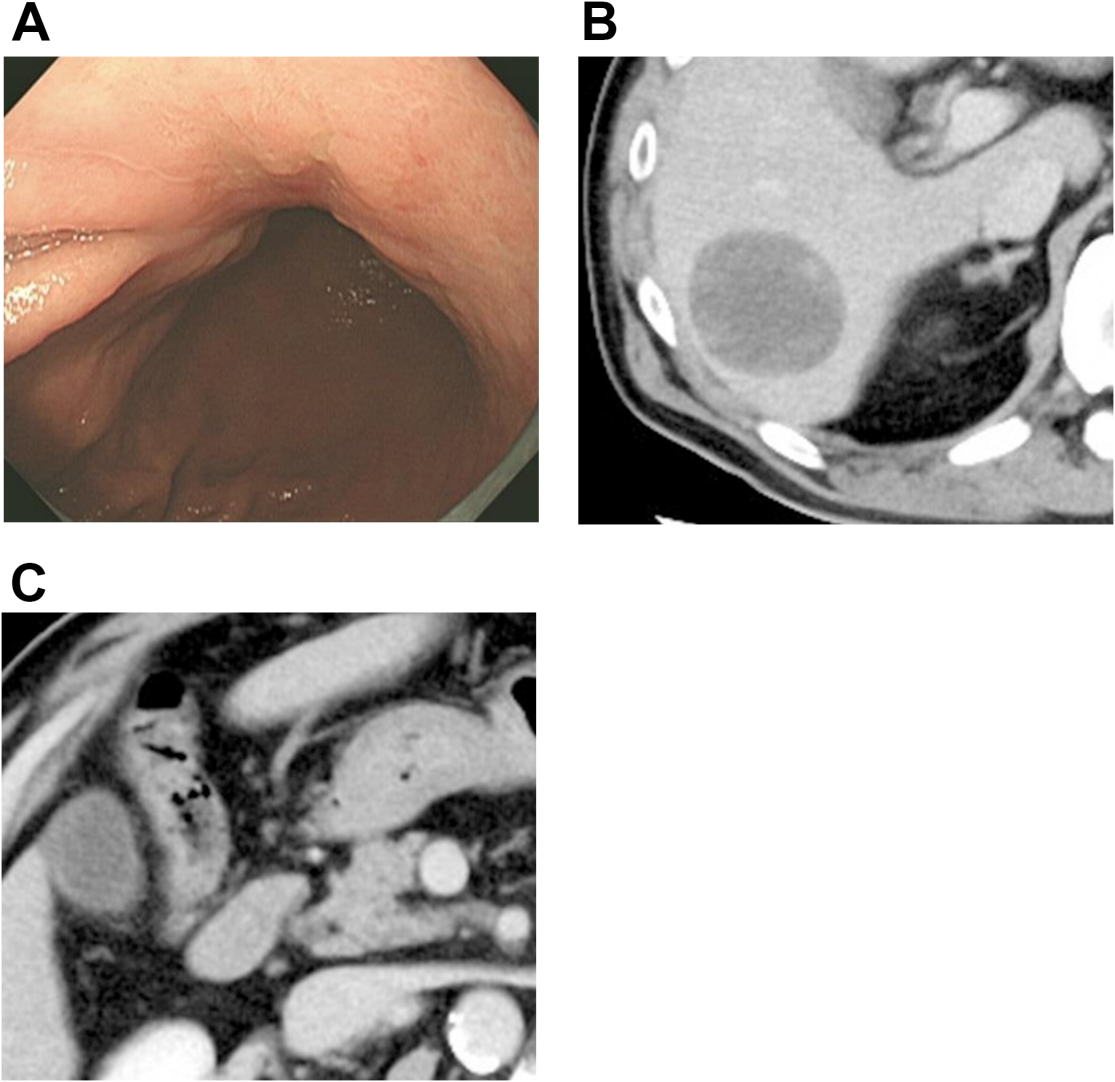
Findings from post-chemotherapy examinations. **(A)** After first-line chemotherapy, the tumor shows marked shrinkage and appears as a scar-like lesion. **(B)** After chemotherapy, the hepatic metastasis has shrunk, exhibiting a smooth surface and nearly homogenous internal characteristics. **(C)** Post-chemotherapy imaging shows a significant reduction in the size of previously enlarged lymph nodes.

The patient received four cycles of SOX (S-1 [80 mg/m²/day] for 14 days plus oxaliplatin [130 mg/m²] every 3 weeks) combined with nivolumab (360 mg every 3 weeks). Post-treatment endoscopy showed marked tumor shrinkage, with the lesion appearing almost scar-like (**Figure 2A**), and CT demonstrated liver metastasis and lymph node shrinkage (**Figures 2B, C**). Distal gastrectomy with D2 lymphadenectomy and partial hepatectomy were performed (**Table 1**).

**Table 1:** Clinical timeline of treatment response.

Date	Clinical event	Procedures/interventions	CEA level (ng/mL)	Imaging findings	Notes
2022/01/12	Initial Consultation	Patient presentation	–	–	–
2022/02/01	Diagnostic Endoscopy	Upper gastrointestinal endoscopy was performed • Location: Lesser curvature of the gastric angle • Size: 6 cm, type 2 tumor	40.01 ng/mL (elevated)	–	Carbohydrate antigen 19–9 within the normal range.
2022/02/02	Diagnostic Imaging	Contrast-enhanced computed tomography (CT) was performed	–	• Regional lymphadenopathy: Multiple enlarged nodes • Hepatic metastasis: 60 × 53 mm lesion in segment 6 with heterogeneous internal structure and irregular margins	Stage IV (M1: hepatic metastasis).
2022/03/03	Neoadjuvant Chemotherapy—Cycle 1	Combination chemotherapy with SOX (S-1 and oxaliplatin) plus nivolumab was administered	42.4 ng/mL (increased 6.0% from baseline)	–	–
2022/04/06	Neoadjuvant Chemotherapy—Cycle 2	Combination chemotherapy with SOX plus nivolumab was administered	11.2 ng/mL (decreased 72.0% from baseline)	–	Marked decline in carcinoembryonic antigen levels.
2022/05/11	Mid-Treatment Assessment	Clinical evaluation was performed	8.7 ng/mL (decreased 78.3% from baseline)	–	Carcinoembryonic antigen levels demonstrated normalization trend.
2022/05/18	Neoadjuvant Chemotherapy—Cycle 3	Combination chemotherapy with SOX plus nivolumab was administered	–	–	–
2022/06/08	Treatment Response Assessment	Tumor marker monitoring was performed	7.2 ng/mL (decreased 82.0% from baseline)	–	Carcinoembryonic antigen levels approached the normal range.
2022/06/15	Neoadjuvant Chemotherapy—Cycle 4 (Final)	Combination chemotherapy with SOX plus nivolumab was administered	–	–	Completion of four cycles of neoadjuvant chemotherapy.
2022/07/04	Post-Chemotherapy Imaging	Contrast-enhanced CT was performed	–	• Hepatic metastasis: 49 × 44 mm (25% size reduction) • Improved margin definition • Homogeneous internal structure	Favorable tumor response; patient met criteria for curative resection.
2022/07/06	Post-Chemotherapy Assessment	Tumor marker monitoring was performed	8.0 ng/mL (decreased 80.0% from baseline, still slightly elevated)	–	Carcinoembryonic antigen levels demonstrated substantial reduction but remained slightly elevated above the normal range (reference: ≤5 ng/mL).
2022/07/22	Post-Chemotherapy Endoscopy	Upper gastrointestinal endoscopy was performed	–	• Primary tumor: Completely scarified • Marked regression or disappearance of visible tumor	Endoscopic complete response (RECIST Grade 2–3 equivalent).
2022/08/01	Definitive Surgery	• Distal gastrectomy with D2 lymphadenectomy • Billroth I reconstruction • Partial hepatic resection	3.0 ng/mL (within normal range)	• Hepatic metastasis: En bloc resection with clear margins	Curative-intent (R0) resection achieved.
2022/08/30	Surgical Pathology Results	Final pathological examination	3.0 ng/mL (within normal range)	• Gastric and hepatic specimen: No residual tumor • Regional lymph nodes: No residual tumor • Hepatic specimen: No residual tumor • Pathological complete response (pCR) confirmed.	–
2022/09/14	Post-Operative Follow-up	Clinical assessment and tumor marker monitoring were performed	3.5 ng/mL (within normal range)	–	Sustained normalization of carcinoembryonic antigen levels.

Chronological summary of diagnostic findings, chemotherapy courses, and surgical outcomes in a gastric cancer patient with hepatic metastasis treated with neoadjuvant SOX + nivolum ab chemotherapy.

CEA, carcinoembryonic antigen; CT, computed tomography; D2, D2 lymphadenectomy; pCR, pathological complete response; SOX, S-1 and oxaliplatin; tub2, moderately differentiated tubular adenocarcinoma; por, poorly differentiated adenocarcinoma.

The postoperative course was uneventful. The patient remained recurrence-free for 3 years and 3 months postoperatively.

## 3 Diagnostic assessment

**Figure 3:**
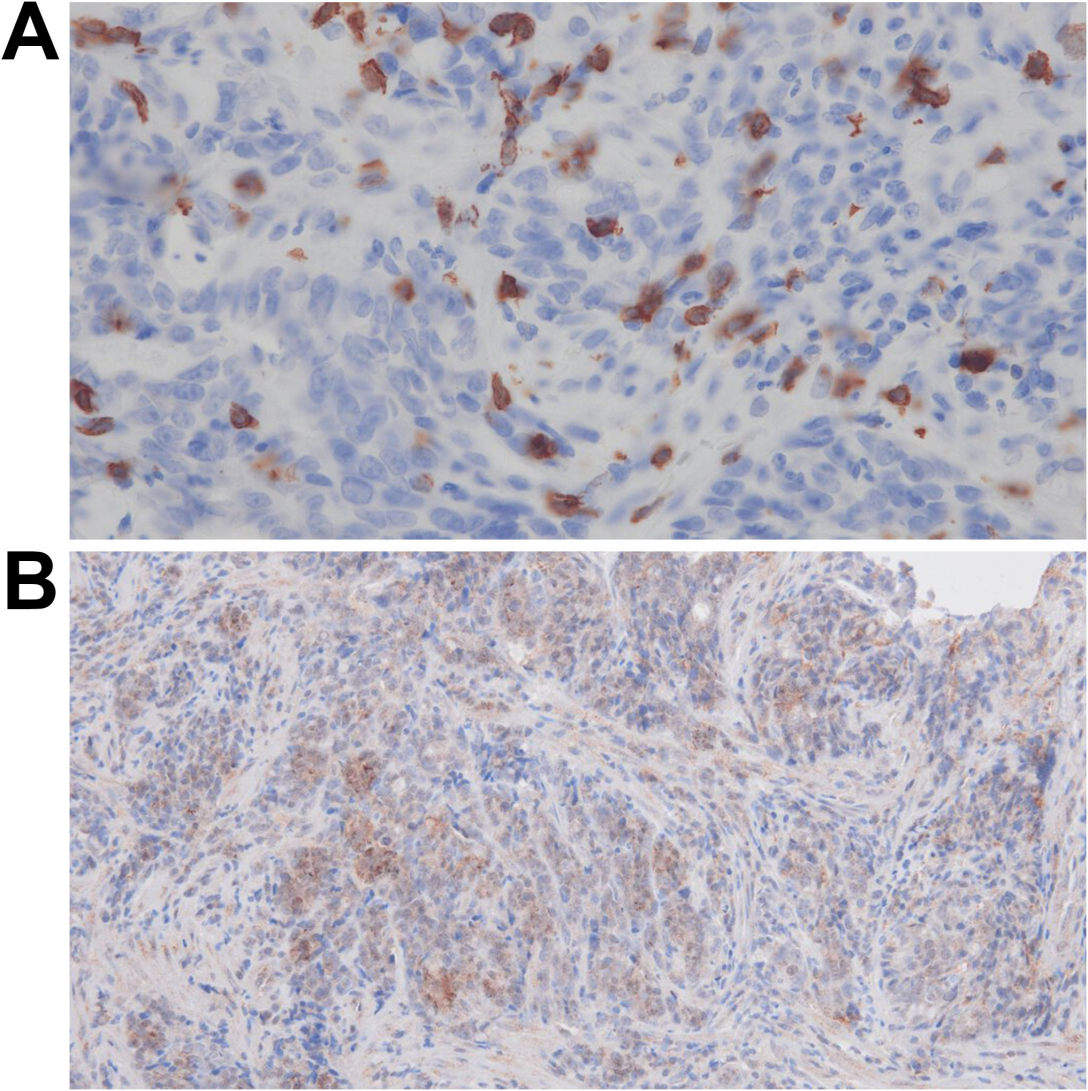
Findings from immunohistochemistry. **(A)** Biopsy specimens stained with anti-CD8 antibody, observed at ×400 magnification. An average of approximately 60 CD8-positive cells per high-power field is observed. **(B)** Biopsy specimens stained with anti-PD-L1 antibody (clone 28-8), observed at ×200 magnification. Numerous PD-L1-positive cells are observed, with a CPS of ≥70%. PD-L1, programmed death-ligand 1; CPS, combined positive score.

Histopathological evaluation revealed complete tumor cell regression in the stomach, lymph nodes, and liver. Immunohistochemistry revealed marked CD8+ cell infiltration. Quantitative assessment was performed at ×400 magnification using 10 randomly selected high-power fields from a tumor-rich biopsy specimen (>90% tumor cellularity; field size: 330.77 × 185.79 μm²). Systematic counting revealed a mean CD8+ tumor-infiltrating lymphocyte (TIL) density of 59.5 ± 14.3 cells/high-power field (HPF) (range: 28–83 cells/HPF; **Figure 3A**), corresponding to approximately 968 cells/mm^2^. For consistency, the CD8^+^ cell counts reported in reference (10) were recalculated to match the HPF area used in our study. Using clone 28-8, a PD-L1 combined positive score (CPS) of ≥70% was observed (**Figure 3B**). Molecular testing confirmed that the tumors were MSS, EBV-negative, and tumor mutational burden (TMB)-low. A germline TP53 mutation (p.R106C) was identified using next-generation sequencing.

## 4 Discussion

This case report presents a patient with unresectable advanced gastric cancer (MSS, EBV-negative, and TMB-low) who achieved pCR following four cycles of SOX plus nivolumab, followed by curative surgery. Histopathology confirmed the absence of viable tumor cells in the primary lesion, lymph nodes, and liver metastases, highlighting the curative potential of immunochemotherapy, even in traditionally less responsive molecular subtypes.

We considered four main hypotheses to explain the marked response to immune checkpoint blockade:

- Presence of a tumor microenvironment that is highly conducive to neoantigen generation.
- Effective blockade of tumor immune escape mechanisms by nivolumab.
- Before chemotherapy, the density of CD8+ TILs was relatively high, despite the tumor being MSS.
- Synergistic chemotherapy and immunotherapy.

The first hypothesis proposes that neoantigens are generated when mutations occur in genes encoding cell surface antigens. These mutations are normally repaired using the DNA mismatch repair (MMR) system. In tumors with defective MMR, these mutations accumulate without repair, leading to the production of abundant neoantigens. The remarkable efficacy of combined chemotherapy and immune checkpoint inhibition in MSI-H gastric cancer has been demonstrated in two pivotal phase III trials: CheckMate 649 (1) and KEYNOTE-859 (2). In both trials, the hazard ratio (HR) was 0.34, indicating an extremely favorable outcome. In this case, pCR was achieved after four cycles of SOX plus nivolumab, initially suggesting an MSI-H tumor. However, subsequent testing revealed an MSS phenotype, making this case academically intriguing. However, because this tumor was MSS, MMR deficiency-related neoantigen accumulation was unlikely.

A high TMB (TMB-H) has been proposed as another cause of increased neoantigen production, in addition to MMR deficiency. TMB has been proposed as a surrogate for neoantigen load and a predictor of response to ICIs in various malignancies (3, 4). According to the NCC Oncopanel, the TMB in our patient was 7.8 mutations per megabase, including both coding and non-coding regions. This value falls below the widely accepted threshold for high TMB (≥10 mut/Mb) and is therefore considered TMB-low. Taken together, these findings suggest that the neoantigen load in this tumor was probably not elevated, despite the exceptional clinical response observed.

The second hypothesis is as follows: A striking feature of this case was the exceedingly high PD-L1 expression. Immunohistochemical staining with the 28–8 clone showed a CPS of ≥70%, a likely contributing factor to the marked response to nivolumab therapy. Although most pivotal clinical trials, including KEYNOTE (2, 5), used the 22C3 clone, multiple comparative studies have demonstrated high concordance between the 22C3 and 28–8 clones across tumor types, including gastric cancer (6, 7). Thus, the markedly elevated CPS observed in this case represents a reliable and biologically significant marker of PD-L1-driven tumorigenicity. PD-L1 expression is independently associated with ICI responsiveness in gastric cancer (8). This case aligns with this trend, despite the absence of traditionally favorable markers, such as high TMB or MSI-H.

In the CheckMate 649 trial, subgroup analyses of overall survival (OS), progression-free survival (PFS), and HRs were conducted according to CPS values (1). However, the highest stratification used in that trial was CPS ≥ 10; analyses for very high CPS levels, such as ≥20, ≥50, or ≥70, were not conducted. Similarly, in the KEYNOTE-859 trial, which evaluated pembrolizumab (2), survival and response outcomes in patients with very high CPS scores were not analyzed. This is likely because the number of patients with extremely high CPS values was minimal. Nevertheless, both the CheckMate 649 and KEYNOTE-859 trials consistently demonstrated that higher CPS values are associated with improved OS, PFS, and HR. Although direct evidence for cases with very high CPS values is currently lacking, the trend of increasing clinical benefits with higher CPS values is well documented. The favorable response observed in the present case is consistent with these results.

The third hypothesis is as follows: CD8+ TIL density was 59.5 ± 14.3 cells/HPF (×400), representing the mean ± standard deviation from 10 randomly selected HPFs. Evaluation of CD8+ TILs varies substantially across studies; some report mean counts of approximately 77.2 cells/HPF in MSS and 100.9 cells/HPF in MSI (9). Although this does not indicate an unusually high number of CD8+ TILs compared with MSI cases, it exceeds the median CD8+ TIL infiltration reported in a Korean cohort of MSI-H gastric cancers (18 cells/HPF in tumor center, 24 cells/HPF in invasive margin) (10), suggesting relatively high cytotoxic T cell infiltration in this MSS tumor.

The fourth hypothesis is as follows: This case raises important questions regarding the mechanistic interplay between chemotherapy and immunotherapy. Oxaliplatin induces immunogenic cell death (11), thereby enhancing tumor antigen presentation and promoting the recruitment of effector T cells. Furthermore, neoadjuvant chemotherapy using the SOX regimen increases the number of TILs in gastric cancer (12). The combination with nivolumab may have synergistically enhanced antitumor immunity, ultimately leading to pCR. Although the precise contribution of each treatment component remains unclear, their collective effect was unequivocally beneficial to this patient. Taken together, the second, third, and fourth hypotheses apply to this study.

The oncopanel revealed a germline TP53 missense mutation (p.R106C) within the protein’s DNA-binding domain. TP53 is a well-characterized tumor suppressor involved in DNA repair, cell cycle arrest, apoptosis, and immune response regulation. Although the clinical significance of this mutation remains uncertain due to conflicting ClinVar interpretations, TP53 mutations have been associated with increased genomic instability and altered immune microenvironments in some cancers (13, 14). However, in the absence of elevated TMB levels and preserved mismatch repair proficiency, the immunological impact of the TP53 mutation in this case was minimal. Nonetheless, its presence underscores the complexity of germline findings in the era of broad-panel sequencing and highlights the need for careful interpretation.

Liang et al. (15) reported the clinical benefits of conversion surgery after first-line treatment with ICIs combined with chemotherapy in patients with stage IV gastric cancer. In their study, a comparison between the conversion surgery and nonsurgery groups revealed a clear survival advantage in the surgery group. The median OS was “not reached” in the surgery group and 19.9 (95% confidence interval [CI]: 17.2–22.5) months in the nonsurgery group; the median PFS was 20.4 (95% CI: 15.6–25.1) versus 8.9 (95% CI: 7.1–10.7) months, demonstrating a significant improvement in both OS and PFS with conversion surgery.

The indications for conversion surgery included evidence of a marked response to immunochemotherapy, such as the disappearance of peritoneal metastases. In the subgroup analysis stratified by PD-L1 CPS, patients with a CPS ≥5 accounted for a high proportion (69%) of the surgery group, whereas those with a CPS <5 accounted for 58.5% of the non-surgery group, resulting in a significant difference in CPS distribution between the two groups (P = 0.003) (15). Our patient had a CPS of approximately 70, which is consistent with these findings.

Regarding the optimal timing of conversion surgery, Yoshida et al. indicated that surgery is generally performed after four to six cycles of immunochemotherapy once a partial or complete response has been achieved, and R0 resection is deemed feasible (16).

By contrast, the benefits of including ICIs in postoperative adjuvant chemotherapy were evaluated in the ATTRACTION-5 trial. The 3-year recurrence-free survival (RFS) rate was 68.4% (95% CI: 63.0%–73.2%) in the nivolumab plus chemotherapy group and 65.3% (95% CI: 59.9%–70.2%) in the placebo plus chemotherapy group. The HR for RFS was 0.90 (95.72% CI: 0.69–1.18; *p =* 0.44), indicating no significant difference between the two groups (17).

Therefore, there is currently no clear evidence to support the inclusion of ICIs in postoperative adjuvant therapy.

Previous case reports have described pathological complete response (pCR) of the primary gastric lesion and lymph nodes in advanced gastric cancer without liver metastasis, achieved without the use of ICIs (18, 19). However, only two reported cases achieved pCR across all disease sites—including the primary tumor, liver metastasis, and lymph node metastases—without ICIs, and both involved HER2-positive gastric cancer treated with trastuzumab combined with cytotoxic chemotherapy (20, 21).

In contrast, the present case involved HER2-negative, microsatellite-stable (MSS), and tumor mutational burden–low gastric cancer, a biological subtype lacking established molecular targeted options. In this context, the achievement of pCR across all disease sites following SOX chemotherapy combined with nivolumab suggests that immune checkpoint inhibition may have played a complementary role beyond that of cytotoxic chemotherapy alone.

The patient has remained disease-free for over 3 years since surgery, suggesting that the antitumor immune response may have provided durable immunological memory. This long-term remission reinforces the concept that selecting MSS gastric cancer patients, particularly those with high PD-L1 expression, can result in substantial and lasting benefits from ICI-based therapy. Although PD-L1 expression alone is not a definitive biomarker, it remains valuable in this context.

This case contributes to the limited but growing body of evidence that ICIs may be effective in gastric cancer, even in the absence of classical predictors such as MSI-H or TMB-high. This emphasizes the potential of the PD-L1 CPS as a standalone predictive biomarker and supports further investigation into combinatorial regimens that exploit both immune and chemotherapeutic mechanisms. This study highlights the importance of individualized molecular profiling for optimizing therapeutic strategies for advanced gastric cancer.

## 5 Limitations

This case report has some limitations. Immunohistochemical analyses of pre-treatment specimens showed high PD-L1 expression (CPS ≥ 70%) and moderate CD8+ TIL infiltration (approximately 60 cells per HPF), comparable to or slightly higher than that typically observed in microsatellite-stable gastric cancers. However, intermediate biopsy samples were not available during treatment. Therefore, dynamic changes in the tumor immune microenvironment, such as variations in immune effector cell density, phenotype, and cytokine activity, could not be evaluated.

The precise immunological mechanisms leading to complete pathological response remain unclear. As this is a single case report, further studies with serial sampling and comprehensive molecular profiling are warranted to clarify the temporal immunological processes underlying exceptional responses to immunochemotherapy.

## 6 Patient perspective

When I was first diagnosed with advanced gastric cancer, I was overwhelmed by my fear and uncertainty about the future. Chemotherapy and immunotherapy were physically and mentally demanding; however, I continued the treatment with strong support from my doctors and family members.

Learning that no viable cancer cells remained after the surgery gave me an indescribable sense of relief and gratitude. However, I was concerned about the possibility of recurrence before each follow-up visit. Every time I heard that there were no signs of the disease, I felt deeply reassured and thankful, and I regained the courage to move forward. I sincerely hope that my experience encourages other patients to face similar challenges.

## Data Availability

The raw data supporting the conclusions of this article will be made available by the authors, without undue reservation.
